# Ambient Pressure Changes as an Unrecognized Risk Factor for Pressure Ulcer Development in Wheelchair Users with Air Cell-Based Seat Cushions

**DOI:** 10.3390/s26134233

**Published:** 2026-07-03

**Authors:** Leon Linder, Heiko Wagner, Klaus Peikenkamp

**Affiliations:** 1 Laboratory for Biomechanics, FH Münster University of Applied Sciences, Bürgerkamp 3, 48565 Steinfurt, Germany; 2 Department of Movement Science, University of Münster, Horstmarer Landweg 62B, 48149 Münster, Germany

**Keywords:** pressure ulcer, air cell seat cushion, ambient pressure, surface pressure, wheelchair, barometric pressure, biomechanics

## Abstract

**Highlights:**

**What are the main findings?**

- Ambient pressure drops increase surface pressure on air-cell-based seat cushion users.
- Mean surface pressure increases of 11–62% were measured across five scenarios.
- Moderate pressure drops (30 hPa) already reduce time to cell death by 16 min; large pressure drops (250 hPa) reduce it by up to 55 min.

**What is the implication of the main finding?**

- Risk category changes occurred in up to 66.7% of all investigated conditions.

**Abstract:**

Air cell-based wheelchair seat cushions are widely used for pressure ulcer prevention in individuals with limited mobility. However, the influence of ambient pressure variations on the mechanical surface pressure acting on users has not been systematically investigated. This study presents a dedicated measurement concept to quantify these effects under controlled laboratory conditions. Surface pressure was quantified using a dual-range FSR sensor calibration, achieving a minimum resolution of 0.0012 N/cm^2^ per digit. Five representative scenarios were investigated, covering ambient pressure reductions between 30 hPa (elevator rides) and 250 hPa (aircraft takeoff). Measurements were conducted across 150 series, combining five initial internal cushion pressures and six load levels. Ambient pressure reductions led to measurable increases in surface pressure across all conditions, ranging from 11.2 ± 11.0% (30 hPa) to 62.0 ± 45.3% (250 hPa). Risk assessment based on a pressure–time cell death model revealed risk category changes in up to 66.7% of all conditions. Mean reductions in time to cell death ranged from 16 min (30 hPa) to 55 min (250 hPa), following a logarithmic relationship (adjusted R^2^ = 0.984). These findings highlight ambient pressure variation as a previously unrecognized influencing factor on pressure ulcer risk in wheelchair users with air cell-based seat cushions.

## 1. Introduction

Pressure ulcers remain one of the most prevalent comorbidities in patients with limited mobility, with reported prevalence rates of approximately 36–50% [1,2,3]. Among wheelchair users, these lesions predominantly occur in the seated area and arise from a multifactorial etiology [4]. Excessive and prolonged mechanical loading during sitting is considered a key contributing factor [5]. While sitting, approximately 50% of body weight is supported by the buttocks and proximal thigh regions, which together account for only about 8% of the total body surface area. As a result, maximum surface pressures Z of approximately 3–5.5 N/cm^2^ can occur [6,7,8,9]. Insufficient pressure redistribution due to restricted mobility leads to sustained mechanical compression as well as shear forces acting on the skin and subcutaneous tissue layers [6,7,8,9]. Consequently, soft tissue is compressed against underlying bony structures, particularly the ischial tuberosities, which may result in localized ischemia. The primary determinants for the development of ischemia-related tissue damage and, ultimately, pressure ulcers are the magnitude of the applied interface pressure and the duration of its exposure [10,11,12].

In clinical practice, two fundamental strategies have been established to reduce the risk of pressure ulcers developing. Each strategy addresses one of the two key contributing factors: pressure magnitude or pressure duration. The first strategy involves regularly repositioning and offloading the seated area to minimize pressure exposure duration. However, this approach requires the affected individual to be able to perform weight shifts independently. For patients with impaired mobility, this is often not feasible, meaning they are dependent on assistance from caregivers. In practice, limited personnel resources and a heavy workload in nursing care pose significant challenges to the consistent implementation of this strategy [13]. The second strategy involves using specialized seat cushions, which typically consist of foam, gel, air cells or individually tailored material combinations. By allowing the body to immerse into the cushion, the effective contact area between the body and the seating surface increases [3,14,15]. This reduces the locally acting surface pressure Z, as force and contact area are inversely related. In clinical practice, air cell-based seat cushions are used by approximately 15.5% to 38.4% of wheelchair users [16,17].

Compared to other cushion types, air cell-based seat cushions offer several advantages. Due to their construction of interconnected air cells, these cushions can conform dynamically to the seated individual’s anatomy, providing effective pressure relief. Multiple studies have demonstrated that air cell-based cushions achieve greater pressure reduction than comparable gel- or foam-based cushions [18,19]. However, using air cell-based seat cushions is also associated with certain risks. In contrast to other cushion types, wheelchair occupants are required to regularly monitor and adjust the internal air pressure Y to ensure adequate surface pressure Z relief. Underinflation or overinflation can negate the intended pressure-relieving effect and increase the mechanical load on the user [20]. Notably, the control of cushion internal pressure Y is not subject to standardized regulations. As a result, manufacturers often provide qualitative and non-standardized recommendations. For example, some manufacturers advise that:

- A manual hand check should be performed to ensure that at least 0.5–1 inch of clearance remains between the seated individual and the cushion base beneath the lowest bony prominence [21];
- The cushion should be subjectively assessed to determine whether it feels too firm or too soft [21];
- A daily hand check should be conducted, without providing a detailed description of how this assessment should be performed [22].

However, from a patient safety perspective, these recommendations are insufficiently standardized and methodologically very coarse. Relying solely on manual hand checks introduces substantial subjectivity and does not provide reliable control over the actual internal pressure Y within the cushion. Particularly in users with impaired sensation or limited mobility, incorrectly adjusted internal pressure Y may not only negate the intended pressure-relieving effect and, in the worst case, lead to increased localized surface pressure Z exposure. In the absence of binding guidelines or standardized assessment protocols, there is a risk that air cell-based cushions will not perform as intended, meaning the risk of pressure ulcer development or recurrence may not be adequately reduced.

Furthermore, it should be considered that the surface pressure Z acting on the patient is not solely influenced by the adjustment or potential leakage of the air cell-based cushion. Changes in ambient pressure W can also alter the effective surface pressure Z acting on the skin and underlying soft tissue. From a physical perspective, ambient pressure acts as a static offset force on the cushion. The surface pressure Z effectively acting on the patient corresponds to the difference between the internal cushion pressure Y and the external ambient pressure W. Any change in this pressure differential shifts the pressure gradient and may temporarily result in either increased or decreased mechanical loading. This consideration highlights that even with a correctly adjusted air cushion, unforeseen external factors can influence surface pressure Z relief. Therefore, a once-daily verification of proper internal pressure Y may be insufficient.

Therefore, short-term variations in ambient pressure W over the course of a day can influence the effective surface pressure Z acting on air cell-based seat cushions. Many of these ambient pressure W changes can be attributed to changes in altitude, as ambient pressure W decreases by approximately 1 hPa per 8 m increase in elevation [23]. During elevator rides in high-rise buildings or cable car journeys, altitude gains of up to 638 m in high-rise buildings [24] or approximately 2000 m in cable cars [25] may occur within a few minutes without the user leaving the wheelchair. A similar situation can be observed during aircraft takeoff, during which the cabin pressure is reduced to approximately 750 hPa within a few minutes [26]. Furthermore, ambient pressure W may also change due to meteorological conditions without any associated change in altitude. For example, strong low-pressure systems can cause ambient pressure W drops of approximately 30–50 hPa within a few hours [27,28]. As a concrete extreme example, Storm Kyrill in Europe in 2007 can be cited, during which ambient pressure W decreases of approximately 50–60 hPa within 12–24 h were reported in Germany [29]. In addition, convective events such as severe thunderstorms can produce short-term ambient pressure W changes of a few hectopascals within several minutes [30,31].

From a physical perspective, ambient pressure W acts as an offset pressure on the air cells of the cushion. The surface pressure Z effectively acting on the skin and underlying soft tissue corresponds to the difference between the internal cushion pressure Y and the external ambient pressure W. Consequently, a change in ambient pressure W Δp of 30–250 hPa results in a corresponding change in the effective surface pressure Z acting on the loaded seating area, depending on the individual’s contact area. These considerations demonstrate that, even without any change in the internal cushion pressure Y, the ambient offset pressure W can vary substantially. Therefore, it is necessary to systematically investigate how such ambient pressure W variations affect surface pressure Z on the skin and soft tissue and what implications they have for the pressure-relieving effectiveness of air cell-based cushions.

Within the scope of the literature review, no studies were identified that specifically investigate the influence of ambient pressure W variations on the biomechanical consequences of air cell-based seat cushions. The existing scientific literature predominantly focuses on the relationship between the internal inflation pressure Y of air-filled seat cushions and the resulting changes in surface pressure Z [20,32]. Key aspects investigated include pressure distribution, immersion depth, and the risk of bottoming out. Other studies compare different cushion types or materials in terms of their surface pressure Z redistribution under constant ambient pressures W, without considering ambient pressure W or altitude changes as independent influencing factors [33,34]. In addition, biomechanical studies have shown that factors such as sitting posture significantly affect surface pressure Z distribution at the seating interface. However, these investigations were likewise conducted under stable ambient pressures W [35]. Indications of the practical relevance of ambient pressure W variations are currently found mainly in technical reports and manufacturer documentation. These sources recommend adjusting air-based seat cushions in response to altitude changes without experimentally quantifying these effects. Although recent technical developments integrate barometric sensing into air cell-based seating systems, they do not yet systematically address the biomechanical implications of varying ambient pressure conditions W for the individual. Overall, there is a clear interdisciplinary research gap at the interface of biomechanics and sensor technology concerning the influence of ambient pressure W variations on the functional performance of air-cell-based seat cushions.

The objective of this study is to quantify the effect of ambient pressure W reductions on the surface pressure Z acting on users of air cell-based seat cushions under controlled laboratory conditions. Since no systematic experimental investigation of this relationship has been identified in the literature to date, this study aims to close this research gap through a quantitative biomechanical characterization. The study focuses primarily on wheelchair users with impaired or absent sensation, such as individuals with spinal cord injury, who are unable to perceive changes in mechanical loading and therefore cannot initiate compensatory weight shifts. This study is explicitly designed as a laboratory investigation and does not constitute a clinical study. The implications of the observed surface pressure Z changes for pressure ulcer risk are discussed on the basis of established biomechanical models and the current literature. A direct clinical validation was not within the scope of this work. All measurements were conducted under static, laboratory-controlled conditions to eliminate confounding effects caused by wheelchair user movement. As some of the ambient pressure W variations reported in the literature are given as ranges, these were reduced to discrete values for experimental investigation. The following ambient pressure W change scenarios were examined:

1. Elevator rides in high-rise buildings, e.g., from ground level to 300 m (≈30 hPa).
2. Weather-related pressure variations that may reach up to 50 hPa over the course of a day.
3. Elevator rides to observation decks, such as the Burj Khalifa platform (0–638 m, ≈64 hPa).
4. Cable car rides in mountainous regions, with altitude increases from 0 to approximately 2000 m (≈200 hPa pressure decrease).
5. Aircraft takeoff, during which the cabin pressure temporarily decreases by approximately 250 hPa.

The selected scenarios cover a representative range of ambient pressure W reductions from 30 hPa, reflecting common everyday conditions such as elevator rides, to 250 hPa, reflecting more extreme but realistic conditions such as aircraft travel. Together, they span the full spectrum of pressure variations likely to be encountered by wheelchair users in daily life. For each scenario, the quantitative increase in surface pressure Z is determined and contextualized using a biomechanical reference model.

## 2. Methodology

Throughout this study, the investigated pressure quantities are consistently referred to as surface pressure Z, ambient pressure W, and internal pressure Y, as introduced in the Introduction. Surface pressure Z denotes the mechanical interface pressure acting between the seated individual and the seat cushion. Ambient pressure W refers to the surrounding barometric pressure of the environment, while internal pressure Y describes the barometric pressure within the air cells of the cushion. To clearly distinguish between mechanical and barometric pressures, ambient pressure W and internal pressure Y are reported in hectopascals (hPa), whereas surface pressure Z is expressed in N/cm^2^.

**Figure 1:**
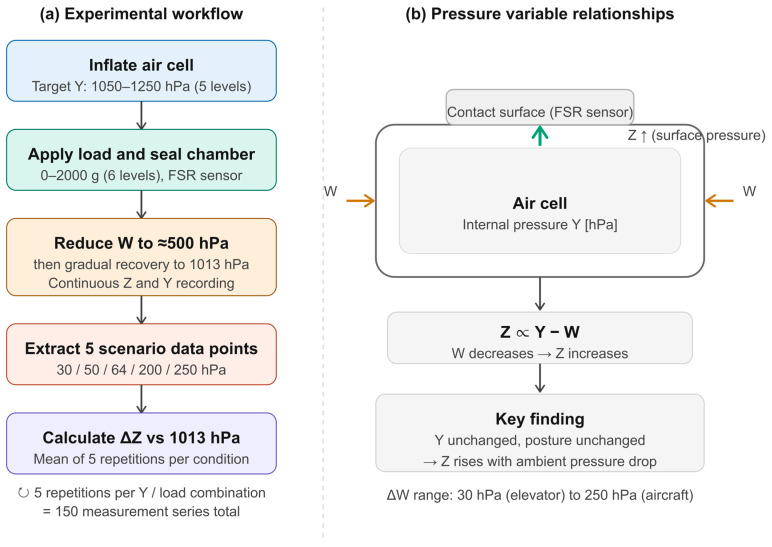
Schematic overview of the experimental procedure. (**a**) Workflow from air cell inflation to data extraction and analysis; black arrows indicate the sequential order of steps. (**b**) Physical relationship between ambient pressure W, internal cushion pressure Y, and surface pressure Z; the green arrow indicates the direction of increasing surface pressure Z, and orange arrows indicate the ambient pressure W acting on the chamber from the surrounding environment. A reduction in W leads to an increase in Z without any change in Y or posture.

To investigate the influence of ambient pressure W on the pressure-relieving performance of air cell-based seat cushions and other air cell-based medical support devices, systematic and reproducible measurement data are required. In order to quantify the effects of ambient pressure W variations, both the surface pressure Z acting on the individual and the prevailing ambient pressure W must be recorded. In addition to these two primary variables, the internal pressure Y of the air cushion is also measured. The measurement of the internal cushion pressure Y serves multiple purposes. First, it allows the direct monitoring of the initial internal pressure Y during the experiments and enables the detection of potential air leakage. The initial internal pressure Y has a substantial influence on the surface pressure Z experienced by the seated individual [20]. Increasing internal pressure Y reduces the deformability of the air cushion and may therefore lead to diminished surface pressure Z reduction. Second, the additional internal pressure Y data allow conclusions to be drawn regarding the behavior of air cushions under changing ambient pressure conditions. All measurements are conducted in a pressure chamber to enable a systematic and reproducible simulation of ambient pressure W variations. A vacuum pump evacuates the air from the pressure chamber containing the air cushion. This allows a wide range of ambient pressure W conditions to be simulated, such as those encountered during aircraft operation (approximately 700–800 hPa). Due to the use of a pressure chamber, surface pressure Z measurements with human subjects are not feasible. Instead, the load that would normally be applied by a patient is simulated using a test rig with discrete load levels ranging from approximately 1 to 20 N. For the experiments, the air cushion is placed in a custom-designed test rig located inside the pressure chamber and subjected to a defined force. After sealing the pressure chamber, the ambient pressure W is reduced to approximately 500 hPa. Following a short stabilization period, ambient pressure W equalization with the external environment is initiated via a fine control valve, causing the ambient pressure W inside the chamber to increase gradually. The restricted flow rate of the valve ensures a slow pressure rise, enabling the acquisition of characteristic curves describing the surface pressure Z between the air cushion and the pressure test rig simulating the patient. Figure 1 provides a schematic overview of the experimental workflow and the underlying physical relationship between the three pressure variables.

To enable a generalized assessment of the effects of ambient pressure W variations, initial internal pressures Y ranging from 1050 to 1250 hPa in increments of 50 hPa were investigated. Applied masses of 0 g (self-weight of the indenter), 100 g, 200 g, 500 g, 1000 g, and 2000 g were used in combination with each pressure level. For each combination of internal pressure Y and applied mass, five repeated measurements were performed, resulting in a total of 150 measurements/characteristic curves available for analysis. For data evaluation, the surface pressure Z acting on the seated individual is first determined for all measurement series corresponding to the five defined scenarios. Mean values are calculated from the five repeated measurements for each combination of initial internal Y pressure and applied force. To facilitate comparison, the percentage deviation of the surface pressure Z observed in each scenario relative to the surface pressure Z at standard ambient pressure (1013 hPa) is subsequently calculated.

To provide a biomechanical context for the quantified surface pressure Z changes, the pressure–time cell death model proposed by Linder-Ganz et al. (2007) [36] is applied as a reference framework. In their publication “Pressure–time cell death threshold for albino rat skeletal muscles as related to pressure sore biomechanics,” Linder-Ganz et al. developed a model grounded in earlier investigations, such as those by Kosiak and others, which describes a pressure–time relationship representing the threshold for cell death in striated skeletal muscle tissue of albino rats. Experimental investigations on pressure-induced tissue damage are frequently conducted using animal models, particularly albino rats, due to the ethical and methodological limitations of performing such experiments directly in humans. The model defines a surface pressure Z–time diagram that characterizes the critical surface pressure Z–time combinations leading to irreversible tissue damage. Based on these investigations, Equation (1) was derived to describe the threshold for cell death:

(1) ZNcm2≥2.31+e0.15t−90+0.9, t min

According to the model, surface pressures Z below 0.9 N/cm^2^ remains below the critical threshold for all exposure times. Surface pressures Z of approximately 3.2 N/cm^2^ corresponds to the upper asymptote of the function and results in a predicted time to cell death approaching 0 min. The surface pressure Z–time relationship was algebraically inverted and combined with the experimentally measured surface pressure Z. This allowed the time to ulceration to be calculated for each scenario and experimental condition at the corresponding pressure level. These calculated exposure times were subsequently used as the basis for a quantitative, model-based indication of potential changes in ulceration risk. It should be acknowledged that the model parameters are derived from albino rat skeletal muscle tissue and results should therefore be interpreted as indicative rather than as direct clinical risk predictions. However, Linder-Ganz and Gefen explicitly connected the animal model to human pressure sore biomechanics through finite element simulations, demonstrating that mechanical stresses in human gluteal muscles during sitting substantially exceed the threshold pressures applied in the rat experiments. The model is therefore considered a conservative reference framework, likely underestimating rather than overestimating risk in human subjects [36].

## 3. Material

For the measurements described in Section 2 to identify the effects of ambient pressure W variations, the following materials were used. The experimental setup is based on a seat cushion manufactured by CICMOD (registered trademark of Osan Electronic (HK) Limited, Hong Kong). The cushion consists of 56 individual square air cushions arranged in a 7 × 8 matrix. Each chamber has an edge length of approximately 5 cm and is made of polyester and thermoplastic polyurethane (TPU). The chambers are interconnected by small air channels. For the purposes of the measurements, a complete seat cushion was not required, so individual air cushions were extracted from the overall matrix. The air cushion used in the experiments was an edge chamber and therefore featured only three air channels. Rubber tubes were inserted into these open channels to allow controlled inflation of the cushion.

The internal barometric pressure Y of the air cushions and the ambient pressure W were measured using pressure sensors of type BMP388 (Bosch Sensortec GmbH, Reutlingen, Germany). These sensors provide a measurement accuracy of ±0.5 hPa and are mounted on breakout boards. Electrical connection to an ESP8266 microcontroller (Espressif Systems, Zhangjiang, China) was established using fine insulated wires. Pressure data were acquired at a sampling rate of 100 Hz via an I^2^C interface and subsequently transmitted to a computer via a wireless LAN connection. For sensor integration into an air cushion, an approximately 2 cm incision was made on the rear side of the chamber. After inserting the sensor, the incision was sealed in the region of the wire feed-through in an airtight manner using a self-adhesive repair foil patch (3-W-Hohenlimburg GmbH, Wietmarschen, Germany) in combination with cyanoacrylate adhesive (3M Company, Saint Paul, MN, USA).

**Figure 2:**
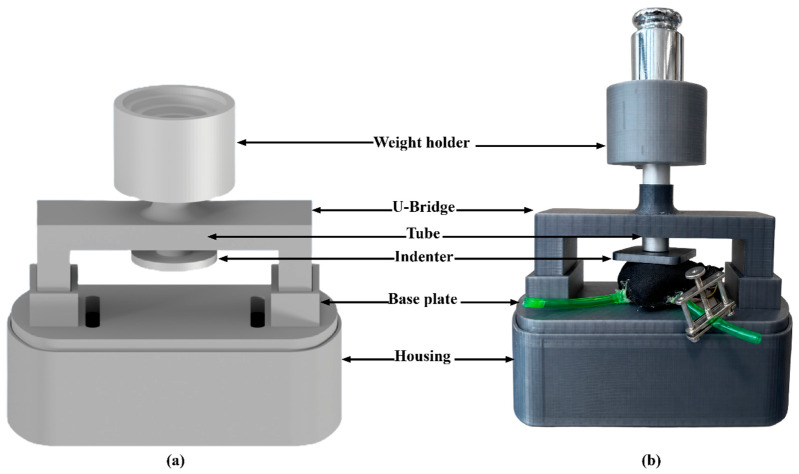
Test rig for surface pressure measurements. (**a**) CAD model showing the individual components. (**b**) Photograph of the assembled test rig with a calibrated test weight placed in the weight holder. The black textile element on the base plate is the extracted air cell under investigation. The FSR surface pressure sensor is mounted on the underside of the indenter and therefore not visible in this view. Green tubes serve as air supply connections to the air cell.

The application of the force, as described in Section 2, was realized using a force test rig specifically designed for the experiments. The test rig is illustrated in Figure 2, showing both a CAD model of the individual components (Figure 2a) and a photograph of the assembled setup (Figure 2b).

The force test rig consists of six individual components which, when assembled, enable a centered and vertically aligned application of force to the air cushion. The lower part of the test rig comprises a base plate that serves both to position the test specimen and to support the U-shaped bridge. The mounted U-shaped bridge features a central bore with an inner diameter slightly larger than that of the rod connecting the weight holder and the indenter. This diameter was selected to minimize friction losses during force transmission while ensuring that the applied load acts orthogonally to the base plate. The indenter has dimensions of 5 × 5 cm, corresponding to the edge length of the air cushions used, thereby ensuring a uniformly distributed load over the cushion surface. The weight holder incorporates several internal radii with increasing depth, which are adapted to the calibrated test weights used (manufacturer G&G GmbH, Kaarst Germany) with an accuracy class of M2. This design ensures that the applied load is transmitted centrally to the rod and, consequently, to the indenter. When applying the loads, the mass of the indenter must also be taken into account, as it contributes to the total force acting on the air cushion. The mass of the indenter is 72 g and was therefore added to each applied test weight. In addition, the test rig includes a housing located beneath the base plate, which contains a battery, the microcontroller with additional electronics, and the ambient pressure sensor. Ventilation slots in the base plate allow cable routing for both the pressure sensor integrated into the air cushion and the sensor used to measure the surface pressure Z between the air cushion and the indenter. Owing to these ventilation openings, the pressure inside the housing equals the ambient pressure surrounding the test rig, allowing the ambient pressure sensor to be located within the housing.

For the measurement of surface pressure Z, force-sensing resistor (FSR) 402 Short Tail sensors manufactured by Interlink Electronics (Fremont, CA, USA) were used. The sensor is a pressure-sensitive resistor whose resistance decreases with increasing applied load, from approximately 10 MΩ in the unloaded state to about 2 kΩ at maximum load. The FSR sensor allows the measurement of forces in the range of 0.2 to 20 N over an active sensing area of 1.267 cm^2^, corresponding to a measurable pressure range of 0.16 to 15.79 N/cm^2^. In addition to the suitable pressure measurement range, the sensor was selected due to its low profile, with a thickness of only 0.46 mm [37].

**Figure 3:**
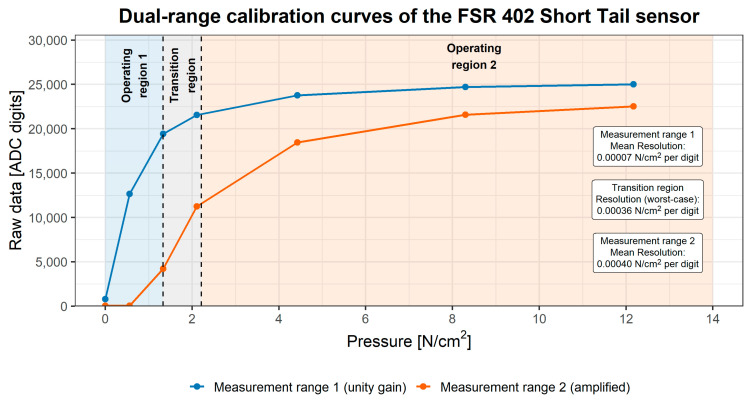
Dual-range calibration curves of the FSR 402 Short Tail sensor. The blue shaded region indicates operating region 1 (below 1.33 N/cm^2^), corresponding to measurement range 1 (unity gain), and the orange shaded region indicates operating region 2 (above 2.21 N/cm^2^), corresponding to measurement range 2 (amplified). The unshaded region between the dashed vertical lines represents the weighted transition region. Mean resolutions are 0.00007 N/cm^2^ per digit (range 1), 0.00036 N/cm^2^ per digit (transition, worst-case), and 0.00040 N/cm^2^ per digit (range 2).

For signal quantification, an external ADS1115 ADC (Texas Instruments Incorporated, Dallas, TX, USA) was employed, providing 16-bit resolution and, as a sigma-delta ADC, reduced quantization noise, improved linearity, and enhanced noise rejection [38]. The FSR sensor was connected to a fixed resistor in a voltage divider configuration. As illustrated by measurement range 1 in Figure 3, which represents the unprocessed FSR sensor output, the output voltage does not increase proportionally with applied pressure. The voltage-force relationship is inherently nonlinear due to the hyperbolic resistance-force characteristic of the FSR element. As the FSR resistance decreases toward its minimum value at higher forces, the incremental voltage change per unit force becomes progressively smaller, resulting in a loss of measurement resolution at higher pressure levels. Consequently, a simple linear calibration is insufficient. To address this, a dual-range signal conditioning circuit was implemented, combining two complementary measurement ranges with a weighted transition region into a single fused calibration, as shown in Figure 3. This approach yields a minimum resolution of 0.0012 N/cm^2^ per digit across the clinically relevant pressure range of 0 to approximately 5.5 N/cm^2^. The detailed circuit design, amplifier parameters, and calibration procedure are provided in Appendix A.

**Figure 4:**
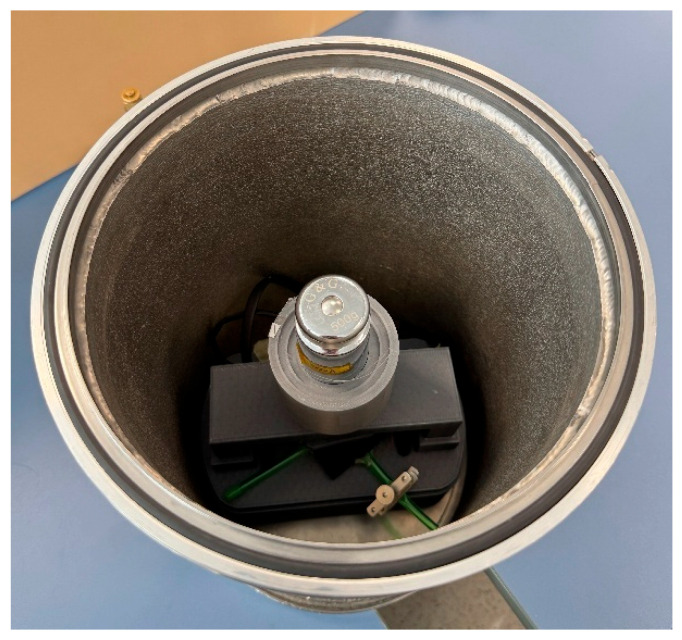
Top view of the experimental setup showing the pressure chamber without lid, the internal test rig with the air cushion in place, and a 500 g test weight applied to the indenter.

To simulate a decreasing ambient pressure W, a vacuum pump of type TRIVAC D 25 B (Leybold GmbH, Cologne, Germany) was used in combination with a vacuum chamber (custom-built). The vacuum chamber is made of steel and has an inner diameter of 20.8 cm and a height of 32.4 cm, corresponding to a total volume of 11 dm^3^. During the measurements, the force test rig with the inserted air cushion and the applied load was placed inside the vacuum chamber. The experimental setup inside the vacuum chamber is illustrated in Figure 4 without the chamber lid. The figure shows the force test rig positioned on the bottom of the vacuum chamber, with the air cushion located on the base plate of the test rig and connected to the green tubes used for inflation and deflation. In addition, a 500 g test weight mounted in the weight holder at the upper end of the indenter is visible.

Using this experimental setup, all 150 measurement series were conducted, comprising the six applied load levels and five initial internal pressure Y settings described above.

## 4. Results

**Figure 5:**
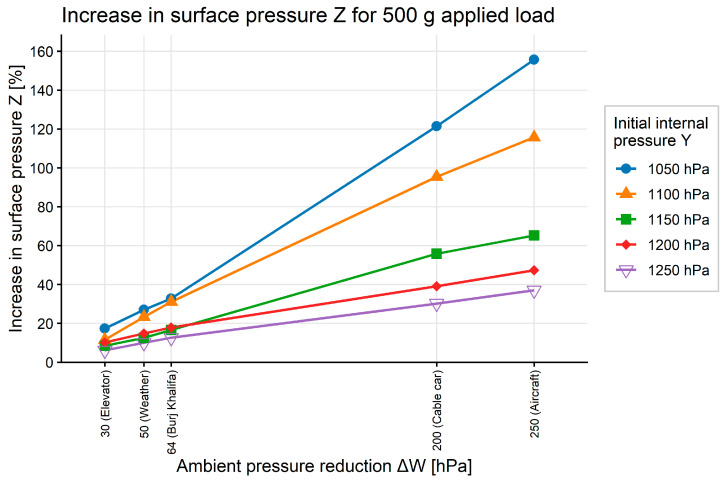
Increase in surface pressure Z as a function of ambient pressure reduction ΔW for an applied load of 500 g, shown for all five initial internal cushion pressures Y. Results for all other load levels showed consistent trends and are provided in Appendix B.

To evaluate the effects of a decrease in ambient pressure W on the surface pressure Z on wheelchair users with air cell-based seat cushions, the results are reported as relative (percentage) changes in surface pressure Z compared to the value measured at the standard ambient pressure W of 1013 hPa for the representative scenarios introduced in the Introduction. For each combination of initial internal pressure Y and applied force, the five repeated measurements were averaged, and the surface pressure Z at standard ambient pressure (1013 hPa), as well as under the five defined scenarios were determined. Across the five repeated measurements per condition, the coefficient of variation was 0.048 ± 0.022. The investigated scenarios include elevator rides in high-rise buildings (300 m ≈ 30 hPa), weather-related pressure variations (50 hPa), elevator rides to observation decks such as the Burj Khalifa platform (0–638 m, ≈64 hPa), cable car rides in mountainous regions (approximately 2000 m ≈ 200 hPa), and aircraft takeoff (approximately 250 hPa). The complete results for all measurement conditions are provided in Table A3 (Appendix B). Figure 5 provides a representative overview of the surface pressure Z increase for an applied load of 500 g across all five initial internal pressures Y. Results for all other load levels showed consistent trends and are provided in Appendix B.

As shown in Figure 5, two distinct trends can be identified in the percentage deviations of the surface pressure Z acting on the seated individual. The first trend represents an increase in surface pressure Z with decreasing ambient pressure W. As expected, a reduction in ambient pressure W leads to an increase in the measured surface pressure Z. This behavior is consistently observed across all rows of the table. The second trend becomes apparent when comparing different initial internal pressures Y at identical applied loads and identical ambient pressure W reductions. In this comparison, a general decreasing trend in the relative surface pressure Z increase can be observed with increasing initial internal pressure Y. In addition, within each group of identical initial internal pressure Y, the surface pressure Z measured at standard ambient pressure does not increase linearly with increasing applied weight. Instead, the relationship between applied load and resulting surface pressure Z shows a non-uniform progression across the investigated weight levels.

**Figure 6:**
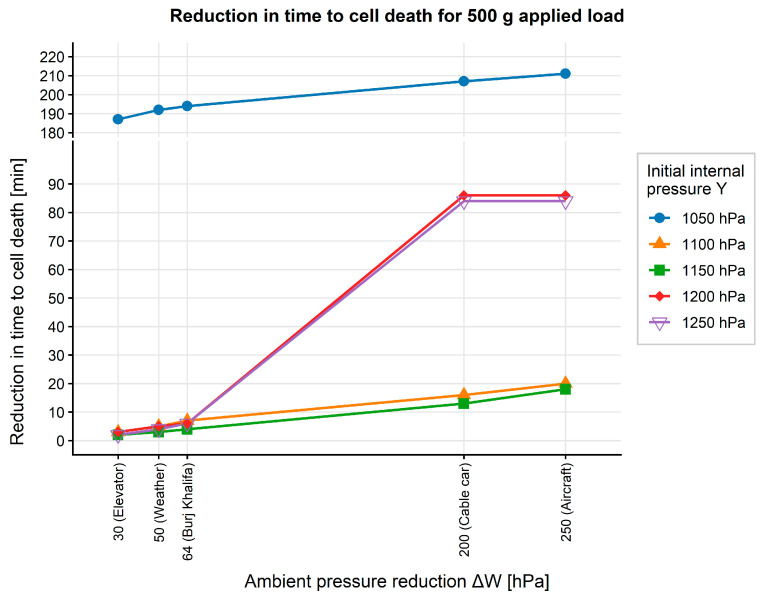
Reduction in time to cell death as a function of ambient pressure reduction ΔW for an applied load of 500 g, calculated based on the pressure–time cell death model proposed by Linder-Ganz et al. (2007) [36], shown for all five initial internal cushion pressures Y. The abrupt increase observed for initial pressures of 1200 and 1250 hPa between 64 hPa and 200 hPa reflects a risk category transition indicating that the ambient pressure-induced surface pressure Z increase exceeds the threshold for predicted immediate cell death at these conditions. For an initial pressure of 1050 hPa, the surface pressure Z at 500 g already exceeds this threshold at standard ambient pressure, resulting in consistently high time reduction values across all scenarios. Complete results for all load levels are provided in Appendix B.

For a model-based indication of potential changes in ulceration risk, the times to cell death were calculated using the pressure–time relationship described in Section 2. The complete results are provided in Table A4 (Appendix B). Figure 6 illustrates the reduction in time to cell death for an applied load of 500 g across all initial internal pressures Y. The abrupt increase observed for initial pressures of 1200 and 1250 hPa between 64 hPa and 200 hPa reflects a risk category transition. At these conditions, the ambient pressure-induced surface pressure Z increase exceeds the threshold for predicted immediate cell death according to the Linder-Ganz model. For an initial pressure of 1050 hPa, the surface pressure Z at 500 g already exceeds this threshold at standard ambient pressure, which is reflected in the consistently high time reduction values across all scenarios. According to this model, the surface pressure Z–time curve exhibits two asymptotic limits. Surface pressures Z below approximately 0.9 N/cm^2^ remain below the critical threshold for all exposure times and are therefore considered safe within the model framework. In contrast, surface pressures Z approaching approximately 3.2 N/cm^2^ correspond to the upper asymptote of the function and result in a predicted time to cell death approaching 0 min. For visualization in Table A4 (Appendix B), these limiting conditions are represented by exposure times of 300 min and 0 min, respectively [36].

To facilitate risk assessment, the times to cell death in Table A4 are color-coded: green (>120 min, low risk), yellow (90–120 min, moderate risk), and red (<90 min, high risk). Conditions exceeding the 3.2 N/cm^2^ immediate cell death threshold are marked with a red border. Measurement conditions 1, 7, 13, and 19 are particularly notable, as under these conditions none of the investigated ambient pressure W changes resulted in surface pressure Z capable of inducing cell death. For these conditions, none of the calculated times to cell death fell below the defined risk thresholds. In contrast, all measured surface pressure Z for measurement condition 30 exceeded the threshold for immediate cell death and, consequently, ulcer formation. For this condition, surface pressure Z levels exceeded the cell death threshold already at standard ambient pressure W, and no further increase in risk due to ambient pressure W reduction was observed. Apart from these five measurement conditions, a reduction in the calculated time to cell death was observed for all remaining conditions. For a total of 20 measurement conditions (2, 3, 4, 5, 6, 10, 11, 12, 15, 16, 17, 18, 21, 22, 23, 24, 25, 27, 28, and 29), at least one change in risk category occurred within a row. In measurement condition 4, a transition across two risk categories was observed. Overall, this indicates that approximately 66% of all measurement conditions exhibited a change in risk category when comparing standard ambient pressure W to the five defined ambient pressure W scenarios. To quantify the risk increase associated with each scenario, three parameters are reported for each case. The first parameter is the mean relative surface pressure Z increase derived from the last row in Table A3 (Appendix B). The second parameter is the mean reduction in time to cell death. For this purpose, the difference in time to cell death between the measurement at standard ambient pressure W (1013 hPa) and the respective scenario condition was calculated for each of the 30 measurement conditions. The resulting time reductions were then averaged across all measurement conditions for each scenario, and the corresponding standard deviation was determined. The individual values used for this calculation are provided in the Table A5 (Appendix B). The third parameter is the number of conditions in which a change in risk category occurred relative to the measurement at standard ambient pressure.

1. **Elevator rides in high-rise buildings (≈30 hPa):**
  Pressure increase: 11.2 ± 11.0%; reduction in time to cell death: 16 ± 46 min; risk category change: 5 measurement conditions (16.67%).
2. **Weather-related pressure variations (≈50 hPa):**
  Pressure increase: 17.5 ± 14.7%; reduction in time to cell death: 29 ± 62 min; risk category change: 7 measurement conditions (23.33%).
3. **Elevator rides to the Burj Khalifa platform (≈64 hPa):**
  Pressure increase: 21.4 ± 17.1%; reduction in time to cell death: 31 ± 64 min; risk category change: 8 measurement conditions (26.67%).
4. **Cable car rides in mountainous regions (≈200 hPa):**
  Pressure increase: 53.8 ± 39.4%; reduction in time to cell death: 49 ± 67 min; risk category change: 18 measurement conditions (60.00%).
5. **Aircraft takeoff (≈250 hPa):**
  Pressure increase: 62.0 ± 45.3%; reduction in time to cell death: 55 ± 67 min; risk category change: 20 measurement conditions (66.67%).

**Figure 7:**
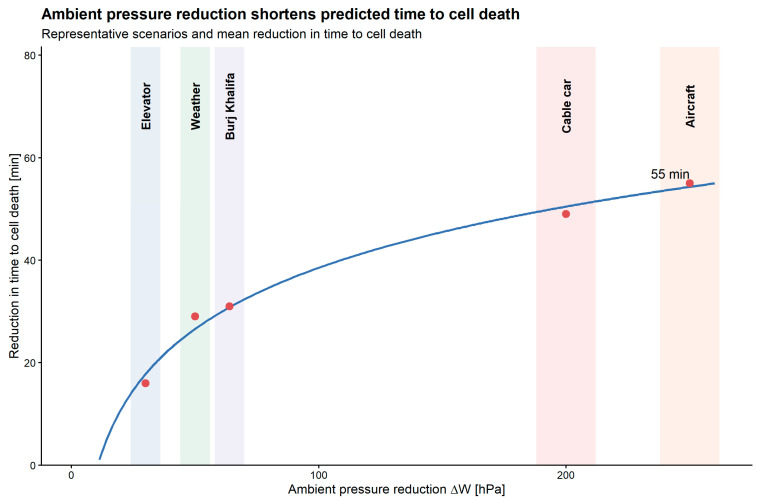
Mean reduction in time to cell death as a function of ambient pressure reduction ΔW for the five investigated scenarios. Blue shaded regions indicate moderate ambient pressure reductions (30–64 hPa), red shaded regions indicate larger reductions (200–250 hPa). The solid line represents a logarithmic trend curve (adjusted R^2^ = 0.984). Red dots indicate the mean reduction in time to cell death for each of the five scenarios.

To provide an aggregated overview of the relationship between ambient pressure reduction ΔW and the resulting mean reduction in time to cell death across all measurement conditions, the results of all five scenarios are summarized in Figure 7. The data points represent the mean reduction in time to cell death for each scenario, as reported above. A logarithmic trend curve was fitted to the five data points (adjusted R^2^ = 0.984), indicating a strong relationship between ambient pressure reduction ΔW and the mean reduction in time to cell death. The trend curve indicates a consistent and monotonically increasing relationship within the investigated range: as ambient pressure reduction ΔW increases, the mean reduction in time to cell death increases accordingly. The concave shape of the curve indicates that the steepest increase in mean reduction in time to cell death occurs at relatively low ambient pressure reductions ΔW, with the rate of increase diminishing progressively beyond approximately 100 hPa. The scenarios associated with moderate ambient pressure reductions ΔW, namely elevator rides and weather-related pressure changes (30–64 hPa), are highlighted in blue/purple/green in Figure 7. These conditions result in mean reductions in time to cell death of 16 to 31 min, as summarized in Table A3 and Table A4 (Appendix B). In contrast, the scenarios associated with larger ambient pressure reductions ΔW, namely cable car rides and aircraft takeoff (200–250 hPa), are highlighted in red/orange and result in mean reductions of 49 to 55 min.

Overall, the results demonstrate that ambient pressure W variations can lead to measurable changes in surface pressure Z and associated ulceration risk across a substantial proportion of the investigated conditions.

## 5. Discussion

In the present study, a dedicated measurement concept and a series of systematic experiments were developed. The aim was to investigate the effects of ambient pressure W variations on surface pressure Z acting on patients using air cell-based wheelchair seat cushions. The surface pressure Z–time injury model proposed by Linder-Ganz, which extends the thresholds originally described by Kosiak [39], was incorporated to estimate the time to predicted cell death. This enabled a model-based assessment of changes in ulceration risk associated with the observed surface pressure Z increases [36].

For an appropriate interpretation of the results, an assessment of the methodological quality of the experimental setup is required. Within the five repeated measurements performed for each individual condition, a low variability was observed, with a coefficient of variation of 0.048 ± 0.022 across all measurements. This indicates good repeatability and supports a positive evaluation of both the measurement concept and the experimental procedure. The measurements yielded reproducible results that reliably reflect changes in surface pressure Z induced by variations in ambient pressure W. The experimental setup itself can also be rated positively. Throughout all measurements, no leakage of the air cushion was observed, even under high pressure gradients between internal pressure Y and external ambient pressure W. This was continuously verified using the pressure sensor integrated into the air cushion. The reproducibility of the results further demonstrates that the force application via the custom-designed force test rig was appropriate and fit for purpose.

The results show that the increase in surface pressure Z caused by a reduction in ambient pressure W becomes smaller as the initial internal pressure Y of the air cushion increases. In other words, cushions with a higher initial internal pressure Y are less sensitive to changes in ambient pressure W. Furthermore, the results indicate that surface pressure Z does not increase proportionally with increasing applied force within a given initial internal pressure Y, but instead exhibits a nonlinear relationship with applied force. The importance of the initial internal pressure Y is illustrated by comparing two measurement series. A series conducted at an initial internal pressure Y of 1150 hPa with an additional weight of 1000 g is compared with a series conducted at 1200 hPa with an additional weight of 200 g. Despite both series exhibiting nearly identical surface pressure Z at standard ambient pressure W (2.05 N/cm^2^ and 2.03 N/cm^2^, respectively), the average increase in surface pressure Z in the 1150 hPa/1000 g condition was 2.15 ± 0.12 times higher than that observed in the 1200 hPa/200 g condition. This example demonstrates that cushions showing comparable surface pressure Z under standard ambient conditions may respond very differently to variations in ambient pressure W depending on their initial internal pressure Y. A plausible explanation for these observations is the deformation behavior of the air cushion. When the applied force increases, the air cushion deforms and the contact area between the indenter and the cushion increases. Because pressure is defined as force divided by contact area, this increase in contact area partially compensates for the increasing force. As a result, the measured surface pressure Z does not rise proportionally with increasing load. In addition, cushions with higher initial internal pressures Y are mechanically stiffer and therefore undergo smaller geometric changes when ambient pressure W varies, which reduces the resulting changes in surface pressure Z.

The aggregated analysis of the five investigated scenarios reveals a clear dependence of the increase in risk of cell death on the magnitude of the ambient pressure W change. With increasing pressure differentials W, both the mean increase in surface pressure Z at the seating interface and consequently the reduction in time to cell death increase, which is also evident in a rising number of risk category transitions. While scenarios involving relatively small ambient pressure W changes, such as elevator rides in high-rise buildings or weather-related pressure fluctuations (≈30–50 hPa), exhibit comparatively moderate mean effects and lead to risk category changes in only a limited proportion of conditions, scenarios associated with larger ambient pressure W variations show a markedly increased sensitivity. At the same time, the results demonstrate that even moderate ambient pressure W changes already induce a remarkable increase in surface pressure Z up to nearly 40% (Table A3) and in the derived risk parameters. This aspect is of particular importance, as the influence of such moderate ambient pressure W variations on the loading conditions experienced by patients has so far received little systematic investigation. Notably, ambient pressure W changes on the order of approximately 200 hPa, as encountered during cable car rides in mountainous regions or during aircraft takeoff, result in pronounced increases in surface pressure Z, a more substantial reduction in time to cell death, and risk category transitions in the majority of the investigated conditions. This observation suggests that beyond a certain threshold of ambient pressure W variation, not only does the mean surface pressure Z increase, but the likelihood of reaching critical surface pressure Z states rises as well. Furthermore, the logarithmic relationship between ambient pressure reduction ΔW and the mean reduction in time to cell death reveals a non-linear risk progression of particular clinical relevance: the steepest increase in risk occurs at comparatively low ambient pressure reductions ΔW, implying that even moderate ambient pressure changes, such as those encountered during elevator rides or weather-related pressure fluctuations, already produce a disproportionately large reduction in time to cell death relative to the magnitude of the pressure change. This finding suggests that the risk associated with ambient pressure W variations cannot be assessed based solely on the magnitude of the pressure change, and that even seemingly minor variations warrant clinical consideration for wheelchair users with air cell-based seat cushions. This assessment is supported by the broader literature on interface pressure and pressure ulcer risk. The Linder-Ganz model was selected for risk assessment in this study because it provides an explicit mathematical formulation of the pressure–time cell death threshold, enabling quantitative calculations rather than relying solely on graphical threshold diagrams. While the model is based on albino rat skeletal muscle data and therefore does not directly translate to human clinical risk, it serves as an established and widely cited reference framework for comparative risk assessment in biomechanical studies. The clinical significance of the observed pressure increases is further contextualized by independent evidence. Gefen et al. demonstrated that sustained tissue deformation can cause cell damage at a microscopic level within minutes, even when clinical signs may not become visible for hours [40]. Furthermore, evidence consistently shows that reductions in interface pressure through appropriate cushion selection are associated with reduced pressure ulcer incidence [2,18,41]. Taken together, the ambient pressure-induced interface pressure increases observed in this study, ranging from 11% to 62% depending on the scenario, suggest potential clinical relevance, particularly for users with impaired sensation who are unable to perceive or compensate for the resulting change in tissue loading. Whether these increases translate into a measurable increase in clinical ulceration risk in humans remains to be confirmed in future studies involving human subjects.

Previous studies on air cell-based seat cushions have predominantly focused on the effects of internal inflation pressure Y, material properties, or posture under constant ambient conditions W [32,33,34,35]. In contrast, the present findings demonstrate that variations in ambient pressure W constitute an additional and previously unaddressed influencing factor that can substantially affect surface pressure Z and derived risk metrics.

The findings of this study have direct implications for the design of smart and adaptive seating systems. Current air cell-based seat cushions are typically inflated to a target internal pressure Y under standard ambient conditions and are not adjusted in response to subsequent changes in ambient pressure W. The results presented here demonstrate that this static approach is insufficient to maintain a stable surface pressure Z across varying environmental conditions, with potential consequences for patient safety in users with impaired sensation.

Future adaptive pressure regulation systems should therefore incorporate continuous ambient pressure W monitoring as an integral component of their sensor architecture. Since barometric sensors are small, cost-effective, and already integrated in several commercially available seating systems for internal pressure Y monitoring, the additional hardware effort for ambient pressure W compensation is minimal. A software-based correction of the internal pressure Y target value triggered by detected ambient pressure W changes would be technically straightforward to implement and could effectively mitigate the surface pressure Z increases identified in this study. Recent feasibility studies have demonstrated that automated interface pressure modulation in SCI patients is technically achievable and well tolerated [42,43], providing a proof of concept for the integration of barometric ambient pressure compensation into such systems. Alternatively, automatic cushion inflation compensation, in which the system actively re-inflates the cushion to restore the target surface pressure Z, represents a more direct control approach that could be realized within existing smart wheelchair technology frameworks [43,44].

From a patient safety perspective, the results highlight that even correctly adjusted cushions may provide insufficient pressure relief under certain everyday conditions such as elevator rides or flights. For wheelchair users with impaired or absent sensation, no compensatory mechanism exists to counteract the ambient pressure-induced surface pressure Z increase, making automatic compensation systems particularly critical for this population. For wheelchair users with intact sensation, the findings suggest that more frequent weight shifts may be required under conditions of significant ambient pressure reduction, since the surface pressure Z increase affects all air cells simultaneously and cannot be offset by redistributing load between cushion zones.

### Limitations

Several limitations of the present study should be acknowledged. First, all experiments were conducted under static laboratory conditions using a rigid indenter to simulate the load applied by a wheelchair user. Human soft tissue exhibits viscoelastic properties, complex geometry, and individual anatomical variability that a rigid indenter cannot replicate. It is therefore not possible to directly transfer the measured surface pressure values to the loading conditions experienced by a specific patient. The direction of this effect is not straightforward: compliant tissue may distribute load over a larger contact area, potentially reducing peak pressures, while at the same time increased tissue deformation under sustained loading may amplify the risk of deep tissue injury.

Additionally, the experiments did not include human subjects, meaning that behavioral responses to increased surface pressure were not captured. In clinical practice, the target population of this study consists primarily of individuals with spinal cord injury and impaired or absent sensation, who are unable to perceive changes in tissue loading and therefore cannot initiate compensatory weight shifts in response to ambient pressure changes. For this population, the passive nature of the experimental setup reflects the actual clinical situation accurately. For wheelchair users with intact sensation, the practical relevance of the observed effects may be reduced, as they are more likely to respond to perceived discomfort through repositioning. However, this does not eliminate the risk entirely, as repositioning frequency and quality are known to be inconsistent even in users with preserved sensation [3,45].

Furthermore, the risk assessment is based on the pressure–time cell death model proposed by Linder-Ganz et al., which was derived from experiments on albino rat skeletal muscle tissue. The threshold values used in the model do not directly correspond to human tissue properties, and the model was not originally developed for clinical risk prediction in wheelchair users. As discussed above, the model was selected because it provides an explicit mathematical formulation enabling quantitative calculations. The calculated times to cell death and risk category changes should therefore be interpreted as indicative rather than predictive. The suggestion of potential clinical relevance, as outlined in the preceding section, is supported by independent evidence from the human pressure ulcer literature [2,40,41] but requires confirmation through dedicated clinical studies. Current clinical guidelines for cushion adjustment and pressure ulcer prevention do not account for ambient pressure variations, suggesting that existing practice does not indirectly compensate for the effects described here.

With regard to the measurement setup, all measurements were conducted using a single air cushion extracted from a commercially available seat cushion matrix. Potential differences between individual cushions due to manufacturing tolerances or material variability could therefore not be assessed. The results should be interpreted as representative of the investigated cushion under the specified conditions. Additionally, the extracted air cell featured only three air channels, which differs from the interconnected multi-cell behavior of a complete cushion. In a full cushion system, pressure equalization between neighboring cells may partially offset the effects observed in the single-cell setup. It should be noted, however, that ambient pressure W acts as a global offset on all air cells simultaneously, regardless of their interconnection. Unlike local loading effects, which may be redistributed between neighboring cells through pressure equalization, an ambient pressure change affects the entire cushion system uniformly. The fundamental effect demonstrated in this study is therefore expected to be representative of complete multi-cell cushion systems, even though the precise magnitude of surface pressure changes may vary depending on cushion geometry and cell interconnection.

Hysteresis effects of the cushion material were not explicitly investigated. As discussed in the methodology, identical internal pressure conditions were restored before each measurement series, and time-dependent viscoelastic deformation was considered unlikely under the chosen experimental conditions. This assumption is supported by the low coefficient of variation observed across repeated measurements. However, under conditions involving prolonged loading, pressure cycling, or defined recovery phases, hysteresis effects may become more pronounced and should be addressed in future studies.

Shear forces were not considered in this study. Interface pressure measurements capture only the normal force component acting on the tissue, whereas shear forces are recognized as an additional contributing factor to pressure ulcer development. According to Gefen et al., shear forces are primarily associated with superficial skin damage, whereas compressive pressure is the dominant contributor to deep tissue injury [40]. Since this study investigates the effect of ambient pressure changes on compressive surface pressure, the omission of shear forces is considered of limited impact on the principal findings. Nevertheless, shear forces may contribute to the overall loading state at the seating interface and their potential interaction with ambient pressure-induced surface pressure changes remains unknown.

Finally, the present study focuses exclusively on mechanical surface pressure and does not capture physiological responses of the skin and subcutaneous microcirculation. A reduction in ambient pressure W leads, via a global decrease in barometric pressure, to a reduced oxygen partial pressure in the inhaled air and may induce systemic hypoxia-related responses that affect vascular tone and blood flow. Although such effects are likely limited under the moderate ambient pressure changes considered here, pressure-induced vasodilation and other microvascular responses may further modulate the tissue response to surface pressure Z in ways not captured by the mechanical analysis. These physiological effects should therefore be considered in future studies aiming for a comprehensive assessment of actual pressure ulcer risk under varying ambient pressure conditions [5,46].

## 6. Conclusions

To our knowledge, this study provides the first systematic quantitative characterization of the influence of ambient barometric pressure variations on the mechanical surface pressure acting on wheelchair users with air cell-based seat cushions. This effect has previously neither been systematically investigated nor considered in clinical practice or cushion management guidelines. The results demonstrate that both the magnitude of the ambient pressure W change and the pre-inflation state of the air cushion have a decisive influence on the increase in surface pressure Z and on the derived risk parameters. It was shown that surface pressure Z measured at standard ambient pressure W alone is insufficient to reliably estimate surface pressure Z increases during a reduction in ambient pressure W, and that the initial internal pressure Y of the air cushion represents an equally relevant influencing factor. These findings provide the first systematic quantitative basis for assessing the biomechanical impact of ambient pressure variations on air cell-based seat cushions and underline the need for future clinical investigations.

The main contributions of this work are threefold. First, it was demonstrated that ambient pressure reductions lead to measurable and clinically potentially relevant increases in surface pressure across all investigated conditions, with mean increases ranging from 11.2% (30 hPa) to 62.0% (250 hPa). Second, both the magnitude of the ambient pressure change and the initial internal cushion pressure were identified as decisive influencing factors, with lower initial pressures resulting in disproportionately larger surface pressure increases. Third, a logarithmic relationship between ambient pressure reduction and mean reduction in time to cell death was identified, indicating that even moderate pressure changes produce disproportionately large effects on the biomechanical risk parameters.

Furthermore, the findings indicate that even moderate ambient pressure W variations can induce measurable effects that have not been considered in previous investigations. As no systematic studies addressing the effects of ambient pressure W changes on air cushion-based seat cushions have been identified in the literature to date, the present work addresses a previously unexplored research gap and provides a foundation for further investigations. Future studies should complement the mechanical insights obtained here with physiological measurements of tissue perfusion and microcirculation in order to further assess the clinical relevance of the observed effects.

## Figures and Tables

**Figure A1:**
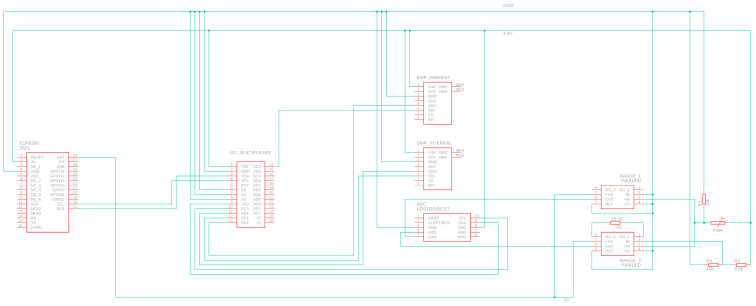
Schematic overview of the signal conditioning circuit. The diagram shows the two BMP388 pressure sensors, the dual-range INA818 instrumentation amplifier configuration, the ADS1115 ADC, the I^2^C multiplexer, and the ESP8266 microcontroller. Auxiliary components such as pull-up resistors and supply decoupling capacitors are omitted for clarity. Red lines indicate component symbols, and teal lines indicate signal connections between components.

## Data Availability

The data presented in this study are available upon request from the corresponding author.

## Appendix A

**Table A1:** Key specifications of the FSR 402 Short Tail sensor (Interlink Electronics) as provided in the manufacturer’s datasheet [37].

Parameter	Value
Active sensing area	1.267 cm^2^ (12.7 mm diameter)
Nominal thickness	0.55 mm
Force sensitivity range	0.1–10.02 N
Corresponding pressure range	0.16–15.79 N/cm^2^
Actuation force	0.1 N
Resistance (unloaded)	~10 MΩ
Resistance (at max load)	~2 kΩ
Force repeatability (single part)	±2%
Force repeatability (part to part)	±6%
Force resolution	Continuous (electronics-dependent)
Hysteresis	+10% (RF+ − RF−)/RF+
Long-term drift	<5% per log_10_(time)
Device rise time	<3 µs
Operating temperature range	−30 to +70 °C
Number of actuations (lifetime)	10 million

**Table A2:** Key specifications of the BMP388 barometric pressure sensor (Bosch Sensortec GmbH) as provided in the manufacturer’s datasheet.

Parameter	Value
Operating pressure range	300–1250 hPa *
Relative accuracy	±8 Pa (equiv. ±0.66 m, 900–1100 hPa, 25–40 °C)
Absolute accuracy	±50 Pa (300–1100 hPa, −20–+65 °C)
Temperature coefficient offset	±0.75 Pa/K (0–55 °C, 700–1100 hPa)
Resolution	0.016 Pa
Digital interface	I^2^C (up to 3.4 MHz)/SPI (up to 10 MHz)
Supply voltage	1.65–3.6 V
Current consumption	3.4 µA at 1 Hz
Operating temperature range	−40–+85 °C
Package dimensions	2.0 × 2.0 × 0.75 mm
Sampling rate (used in study)	100 Hz

* The upper limit of the sensor’s specified operating range (1250 hPa) coincides with the maximum initial internal cushion pressure investigated in this study. This elevated pressure level was used exclusively for leakage control prior to each measurement series. During the actual measurements, internal cushion pressure Y remained within 1050–1250 hPa, with values at 1250 hPa representing the initial state before load application. No measurement anomalies or saturation effects were observed at this pressure level, consistent with the low coefficient of variation across repeated measurements.

The schematic overview of the signal conditioning circuit is shown in Figure A1. The circuit comprises two BMP388 barometric pressure sensors for measuring internal cushion pressure Y and ambient pressure W respectively, two INA818 instrumentation amplifiers implementing measurement range 1 (unity gain) and measurement range 2 (offset subtraction and amplification), an ADS1115 analog-to-digital converter, an I^2^C multiplexer for sensor addressing, and an ESP8266 microcontroller for data acquisition and wireless transmission. For clarity, auxiliary circuitry including pull-up resistors for I^2^C communication and supply decoupling capacitors for noise suppression is omittedin the schematic.

To address the resolution limitation at higher pressures, two separate measurement ranges were realized using two dedicated instrumentation amplifier circuits, both receiving the same FSR voltage divider signal. For measurement range 1, the amplifier operates at unity gain, passing the raw voltage divider output without amplification. This yields a steeply rising characteristic at low pressures and thus high resolution in the low-pressure region, but the output progressively saturates as the FSR resistance approaches its minimum value (blue curve in Figure 3). For measurement range 2, the instrumentation amplifier subtracts a fixed offset voltage of approximately 1.6 V from the FSR signal at its inverting input and amplifies the resulting difference by a factor of approximately 3.4. The offset voltage was selected slightly below the saturation onset of measurement range 1 to ensure an overlap region between both measurement ranges, and the amplification factor was chosen to stretch the remaining signal range across the full ADC input range. This offset subtraction suppresses the low-pressure portion of the signal, where the difference would be near zero or negative, while the subsequent amplification effectively stretches the remaining signal across the full ADC input range. As a result, measurement range 2 provides substantially improved resolution in the higher pressure region where range 1 saturates (orange curve in Figure 3). Both amplifier outputs were digitized simultaneously by the ADS1115, allowing continuous acquisition of both measurement ranges.

As shown in Figure 3, the two measurement ranges exhibit complementary resolution characteristics across the relevant pressure interval. Measurement range 1 provides substantially higher resolution in the low-pressure region below approximately 1.33 N/cm^2^, where the steep slope of the unity-gain amplifier output translates into a large number of ADC digits per unit pressure. However, as pressure increases beyond this point, the FSR voltage divider output approaches saturation, causing the slope of the range 1 calibration curve to flatten progressively. In contrast, measurement range 2 does not yield reliable measurements below approximately 1.33 N/cm^2^, since the FSR signal has not yet exceeded the 1.6 V offset threshold required to produce a meaningful amplifier output. Above approximately 2.21 N/cm^2^, however, range 2 provides a well-resolved and consistent characteristic due to the applied amplification, while range 1 continues to lose resolution. To combine the advantages of both ranges and achieve high measurement resolution across the full pressure interval of interest, the two calibration curves were merged into a single fused calibration using a dual-range fusion approach with a weighted transition in the overlap region.

During range fusion, the fused pressure value is derived exclusively from measurement range 1 ($Z_{1}$, blue curve) for surface pressures Z below 1.33 N/cm^2^, where this range provides its highest resolution. For surface pressures Z above 2.21 N/cm^2^, the fused value is derived exclusively from measurement range 2 ($Z_{2}$, orange curve), which remains well within its reliable operating region. In the intermediate transition interval 1.33≤Z≤2.21 N/cm^2^, both ranges yield valid and overlapping measurements. In this region, the fused pressure value is obtained as a weighted linear combination of both ranges according to Equation (A1), ensuring a continuous and smooth transition without discontinuities at the range boundaries.

(A1) $$Z_{\mathrm{fused}}=w\left(Z\right)\;Z_{1}+\left[1-w\left(Z\right)\right]\;Z_{2}$$

The weighting factor $w\left(Z\right)$ decreases monotonically from 1 to 0 across the transition interval, gradually shifting the contribution from range 1 to range 2 as pressure increases. The lower boundary of the transition region was defined as the pressure level at which range 2 first yields reliable measurements; the upper boundary corresponds to the pressure level at which the resolution of range 1 begins to noticeably decrease due to saturation. This dual-range fusion approach yields a minimum resolution of 0.0012 N/cm^2^ per digit across the clinically relevant surface pressure range of 0 to approximately 5.5 N/cm^2^, as determined from the calibration lookup table. The mean resolution values for each individual measurement range and the transition region are summarized in Figure 3.

## Appendix B

**Table A3:** Results of surface pressure Z increase induced by ambient pressure W changes for the five investigated scenarios (elevator ride: 30 hPa, weather-related pressure change: 50 hPa, Burj Khalifa elevator ride: 64 hPa, mountain cable car: 200 hPa, aircraft takeoff: 250 hPa). The surface pressure Z increase is reported as the percentage change relative to the pressure measured at standard ambient pressure W for all measurement conditions.

Initial Pressure Y [hPa]	Applied Surface Pressure Z at 1013 hPa [N/cm^2^]	Mass [g]	Relative Change to the Applied Surface Pressure Z at 1013 hPa for an Ambient Pressure W Decrease of [%]				
			30 hPa	50 hPa	64 hPa	200 hPa	250 hPa
1050	0.42	0	36.6	45.8	48.6	62.9	63.1
1050	0.53	100	51.1	69.0	80.6	139.7	148.5
1050	0.60	200	37.4	56.9	70.1	180.9	200.6
1050	0.83	500	17.3	27.0	32.8	121.4	155.7
1050	1.01	1000	9.9	16.6	20.7	104.7	133.4
1050	1.97	2000	7.4	11.6	15.0	44.0	54.5
1100	0.69	0	7.2	10.4	11.1	14.1	13.2
1100	1.00	100	11.0	17.9	20.8	40.9	45.8
1100	1.08	200	16.8	24.3	30.0	79.8	85.2
1100	1.21	500	11.5	23.3	31.1	95.4	115.7
1100	1.53	1000	15.4	24.2	28.9	79.2	93.3
1100	2.20	2000	6.7	11.6	14.2	40.4	49.9
1150	0.70	0	2.4	3.6	4.1	5.3	5.4
1150	1.34	100	6.3	8.4	9.3	15.7	13.2
1150	1.71	200	8.2	13.0	15.2	28.3	30.8
1150	1.80	500	8.5	12.5	16.6	55.8	65.2
1150	2.05	1000	8.2	14.5	18.3	49.7	59.4
1150	2.64	2000	5.3	9.7	11.9	34.2	43.1
1200	0.55	0	1.1	8.0	11.2	32.3	39.9
1200	1.44	100	5.0	8.6	11.2	34.9	39.2
1200	2.03	200	4.0	6.8	7.8	22.2	29.5
1200	2.37	500	10.2	14.8	17.9	39.1	47.3
1200	2.60	1000	6.6	11.4	14.2	37.5	45.4
1200	3.14	2000	4.3	7.0	8.8	29.1	34.9
1250	0.87	0	5.5	15.5	24.0	66.4	60.9
1250	1.37	100	7.3	11.9	16.7	38.0	42.2
1250	2.02	200	6.5	9.3	10.9	20.5	22.8
1250	2.55	500	6.1	10.0	12.6	30.2	37.0
1250	2.65	1000	8.3	14.2	17.6	43.3	50.6
1250	3.20	2000	4.4	7.6	10.1	28.3	33.5
Mean ± sd			11.2 ± 11.0	17.5 ± 14.7	21.4 ± 17.1	53.8 ± 39.4	62.0 ± 45.3

**Table A4:** Calculated time to cell death according to the Linder-Ganz model. Color coding: green (>120 min, low risk), yellow (90–120 min, moderate risk), red (<90 min, high risk). A red border indicates conditions where surface pressure Z exceeds the 3.2 N/cm^2^ immediate cell death threshold.

Measurement Condition	Initial Pressure [hPa]	Mass [g]	Applied Pressure at 1013 hPa [N/cm^2^]	Risk Diagram for the Time to Cell Death Based on the Linder-Ganz [min]					
				0 hPa	30 hPa	50 hPa	64 hPa	200 hPa	250 hPa
1	1050	0	0.42	300	300	300	300	300	300
2	1050	100	0.53	300	300	132	114	101	100
3	1050	200	0.60	300	300	116	109	94	93
4	1050	500	0.83	300	113	108	106	93	89
5	1050	1000	1.01	110	105	103	102	90	86
6	1050	2000	1.97	91	89	88	88	79	73
7	1100	0	0.69	300	300	300	300	300	300
8	1100	100	1.00	110	105	103	102	98	98
9	1100	200	1.08	106	101	100	98	91	91
10	1100	500	1.21	102	99	97	96	86	83
11	1100	1000	1.53	97	93	92	91	81	76
12	1100	2000	2.20	88	86	85	84	70	0
13	1150	0	0.70	300	300	300	300	300	300
14	1150	100	1.34	100	98	98	98	97	97
15	1150	200	1.71	94	92	91	91	88	88
16	1150	500	1.80	93	91	90	89	80	75
17	1150	1000	2.05	90	88	87	86	71	0
18	1150	2000	2.64	83	80	78	76	0	0
19	1200	0	0.55	300	300	300	300	300	300
20	1200	100	1.44	98	97	96	96	91	91
21	1200	200	2.03	90	89	89	88	85	83
22	1200	500	2.37	86	83	81	80	0	0
23	1200	1000	2.60	83	80	77	75	0	0
24	1200	2000	3.14	66	0	0	0	0	0
25	1250	0	0.87	300	121	110	106	98	98
26	1250	100	1.37	99	97	96	96	92	91
27	1250	200	2.02	90	89	88	88	85	85
28	1250	500	2.55	84	81	80	78	0	0
29	1250	1000	2.65	82	78	74	69	0	0
30	1250	2000	3.20	0	0	0	0	0	0

**Table A5:** Reduction in time to cell death relative to standard ambient pressure (1013 hPa) for all measurement conditions and investigated scenarios, calculated based on the Linder-Ganz model.

Initial Pressure [hPa]	Weight [g]	Applied Pressure at 1013 hPa [N/cm^2^]	Difference in Time to cell Death Relative to 1013 hPa Based on the Linder-Ganz Risk Diagram [min]				
			30 hPa	50 hPa	64 hPa	200 hPa	250 hPa
1050	0	0.42	0	0	0	0	0
1050	100	0.53	0	168	186	199	200
1050	200	0.60	0	184	191	206	207
1050	500	0.83	187	192	194	207	211
1050	1000	1.01	5	7	8	20	24
1050	2000	1.97	2	3	3	12	18
1100	0	0.69	0	0	0	0	0
1100	100	1.00	5	7	8	12	13
1100	200	1.08	5	7	8	15	16
1100	500	1.21	3	5	7	16	20
1100	1000	1.53	3	5	6	16	21
1100	2000	2.20	2	3	4	18	88
1150	0	0.70	0	0	0	0	0
1150	100	1.34	1	2	2	3	3
1150	200	1.71	2	3	3	6	6
1150	500	1.80	2	3	4	13	18
1150	1000	2.05	2	4	5	19	90
1150	2000	2.64	2	5	7	83	83
1200	0	0.55	0	0	0	0	0
1200	100	1.44	1	2	2	7	7
1200	200	2.03	1	2	2	5	8
1200	500	2.37	3	5	6	86	86
1200	1000	2.60	3	6	8	83	83
1200	2000	3.14	66	66	66	66	66
1250	0	0.87	179	190	194	202	202
1250	100	1.37	2	3	4	7	8
1250	200	2.02	2	2	3	5	6
1250	500	2.55	2	4	6	84	84
1250	1000	2.65	4	9	14	82	82
1250	2000	3.20	0	0	0	0	0
			16 ± 46	29 ± 62	31 ± 64	49± 67	55 ± 67
